# Evaluation of the Standardization in Semen Analysis Performance According to the WHO Protocols Among Laboratories in Tehran, Iran

**DOI:** 10.30699/IJP.14.2.142

**Published:** 2019-06-10

**Authors:** Masha Ahadi, Fereshte Aliakbari, Saeedeh Latifi, Seyed Jalil Hosseini, Atossa Gharib, Abolfazl Movafagh, Zahra Abdolalian, Arash Dehghan, Arsham Moradi, Behrang Kazeminejad, Azadeh Rakhshan, Elena Jamali, Farzad Allameh, Afshin Moradi

**Affiliations:** 1 *Infertility & Reproductive Health Research Center, Shahid Beheshti University of Medical Sciences, Tehran, Iran*; 2 * Department of Medical Genetics, School of Medicine, Shahid Beheshti University of Medical Sciences, Tehran, Iran*; 3 *Department of Pathology, Hamedan University of Medical Sciences, Hamedan, Iran*

**Keywords:** Quality control, Semen analysis, Andrology, Sperm count, Medical laboratory

## Abstract

**Background and Objective:**

Infertility refers to the failure in achieving pregnancy of a couple after one year of regular sexual intercourse without using a protection method. The purpose of this research work was to evaluate the current status of the test and quality control performance in semen analysis in selected laboratories.

**Material and Methods:**

The semen analysis was performed in the Laboratory of Andrology in terms of macroscopic examination which include volume, color, viscosity, pH and acidity, and in terms of microscopy: the rate of sperm movement, the exact number of sperms per ml of semen, the percentage of sperm viability and movement, the presence of germ cells and white blood cells. Several questions for each part of the test were selected and answered by the director of the laboratories or andrology section supervisor.

**Results:**

There was a wide range in the performance of selected medical laboratories in Tehran regarding the standards of semen analysis according to the World Health Organization (WHO) Laboratory Manual for the examination and processing of human semen, fifth edition in 2010. They followed the instructions related to the sample collection in about 70% of the evaluated parameters, initial macroscopic examination in about 87% of the selected subjects, and the microscopic evaluation of sperm in about 65% of the test parameters.

**Conclusion:**

some laboratories do not follow the instructions of the WHO in performing semen analysis, and most of them do not follow the suggested methods in all parts of the test.

## Introduction

Infertility is defined as a global health issue with physical, psychological and social impacts. Infertility refers to the failure in achieving pregnancy of a couple after one year of regular sexual intercourse without using a protection method. In general, from each six couples, one of them experiences a primary or secondary infertility (1,2). Therefore, infertility as an important part of the clinical practice of physicians is seen in 10 - 15% of couples (1,3).

According to the WHO statistics, about 40% of infertility causes is related to the male factors. Semen analysis is usually the first lab test and one of the most important aspects of fertility tracking. The main implication of semen analysis is to determine the fertility status of men (4), and doing a spermogram is among the first steps in the evaluation of infertile couples (5). The indexes that are usually an integral part of any semen analysis include sperm count, movement of sperm, motionless sperm, the number of sperm with normal shape, volume and color of semen, the consistency of semen, pH and the number of white cells (6,7).

The semen analysis measures the production of semen in men, as well as the quality and quantity of sperm. The reported parameters may be different in each lab based on the standards in performing the tests in that lab (8). Furthermore, quality control measures in semen analysis is essential to obtain reliable results in andrology laboratory, and we need to use appropriate quality control and quality assurance methods (9). Moreover, the variability in the values obtained from the sperm analysis among different laboratories indicates the importance of using quality control programs and standard methods. Each lab must have a quality assurance program to ensure that the results are accurate (10).

The basic parameters of sperm analysis, including concentration, morphology and sperm motility, should be examined and regulated by the internal and external quality control measures (10). For instance, in an external quality control program, the tests should be performed on a sample with a well-known parameter by different laboratories and comparing their results (11).

If the results of several measurements of a parameter are close together, the test is reliable (12). Therefore, quality control is necessary to detect and correct systematic and random errors and to modify performance methods of the tests. By standardizing protocols and methods in laboratories, it is possible to reduce the inter-laboratory variation (13). To achieve this purpose, doing the test according to the last edition of the "World Health Organization's Laboratory Manual on the testing and processing of human sperm" is recommended. However; the differences in the results obtained from the seminal fluid analysis of a sample among different laboratories indicates the value of using quality control programs and standardizing methods (14). If there is no proper quality control for this test, the true cause of infertility of the couple may remain undiagnosed and sometimes a wrong diagnostic or treatment decision is made (12).

The aim of this study was to evaluate the current status of the test and quality control performance in semen analysis in the selected laboratories.

## Materials and Methods

With the coordination of the head office that supervised laboratories affiliated to Shahid Beheshti University of Medical Sciences, Tehran, the checklist for procedures of the spermogramme was filled by our team member through an interview with the head of the lab and andrology section in charge of the test in the selected medical laboratories. According to the prepared draft, the equipment, materials and methods, and the documents were evaluated.

**Statistical analysis**

Descriptive statistics were used to analyze the data in this project. 

## Results

All of the surveyed labs followed the instructions regarding the time of sexual abstinence before the sample collection, measurement of the sample volume, making a wet preparation, sperm agglutination report, and evaluating the presence of other cell components.

The majority (70%-99%) of the participants were instructed about reporting the missing part of the specimen, delivery time, time to repeat the test, and doing measurement of the viscosity, reporting the properties of the semen fluid, doing the measurement of the pH, sperm adhesion grading, and performing antibody testing.

Over half (50%-70%) of surveyed labs reported that they keep semen in the incubator at 37°C, determine sperm morphology, and do the standard stain methods to evaluate sperm morphology.

Of the study labs, less than half (20%-50%) asked the patient to urinate before the sample collection, evaluated sperm vitality, and used haemocytometer chambers to do counting the sperms.

Just a small number (0%-20%) of those surveyed labs indicated that they recommend using silastic condoms to the patients if they want to collect the specimen during intercourse and do measure sperm motility estimates in replicate counts.

All data was displayed as following; Table 1 demonstrates the factors related to the sample collection, Table 2 illustrates initial macroscopic examination, and finally Table 3 represents evaluation of the sperm microscopy.

**Table 1:** Factors related to the sample collection

Laboratory standard	Number (n:40)	Percent (%100)
Time of sexual abstinence before the test	40	% 100
Inform the laboratory if the man missed a part of the specimen during the collection or transport	37	% 92.5
Urination before the semen sample collection	17	% 42.5
Deliver the sample to the laboratory within 1 hour of collection (Collection of semen at home)	39	% 97.5
Report on the location of the specimen collection (home or laboratory)	25	% 62.5
Recommend using SILASTIC condoms(In case of the collection of semen by condom during sexual intercourse only in exceptional circumstances)	0	%0
Time to repeat the test (2 to 7 days)	39	% 97.5

**Table 2:** Initial macroscopic examination factors

Laboratory standard	Number (n: 40)	Percent (% 100)
Keep semen in the incubator at 37°C	26	%65
Record the time to convert semen from gel to liquid (15-60 min)	39	% 97.5
Measurement of semen fluid by microscopy	40	٪ 100
Use of the standard method for semen liquid (pipetting and enzymatic method)	16	%40
Estimate of the viscosity of semen	37	%92.5
Measurement of the viscosity of semen by standard method (pipette, Anse, Syringe, Applicator)	33	% 82.5
Reporting the properties of the semen fluid (color, homogeneity, volume, pH)	39	% 97.5
Measure the volume of the specimen by a syringe or graduated cylinder	40	% 100
Measurement of the pH with tornasol paper	39	%97.5
Homogenize the sample	40	%100

**Table 3:** Factors in microscopic investigation of semen

Laboratory standard	Number (n: 40)	Percent(% 100)
Making a wet preparation	40	% 100
Sperm Agglutination Report	40	% 100
Sperm adhesion grading report (few, moderate, many)	30	%70
Reporting other cell components (epithelial cells, leukocytes and immature germ cells)	40	% 100
Sperm motility estimates in replicate counts (taking two aliquots from the semen sample, make two preparations)	0	%0
Sperm vitality (Dye, or by using hypo-osmotic swelling)	8	%20
CASA method for counting sperm	19	%47.5
Haemocytometer chambers	19	% 47/5
Determination of sperm morphology	25	% 62.5
Standard coloring methods (diff quick, Giemsa, SHORR)	23	% 57.5
Perform antibody testing	38	%95

## Discussion

In the current study we evaluated quality control of semen analysis in a selected group of laboratories affiliated to Shahid Beheshti University of Medical Sciences in Tehran. Physicians practicing in the field of infertility usually believe that there is a considerable inter and intra laboratory variation in the test values that sometimes make a correct medical decision difficult or impossible (11). 

To the best of our knowledge, this is the first study that evaluated standards of semen analysis in Iran. There is a wide range in the performance of selected Tehran medical laboratories regarding the standards of semen analysis according to the WHO laboratory manual for the examination and processing of human semen, fifth edition in 2010. They followed the instructions related to the sample collection in about 70% of the evaluated parameters, initial macroscopic examination in about 87% of the selected subjects, and the evaluation of sperm microscopy in about 65% of the test parameters.

Filimberti et al., documented the quality control issue and conducted a survey in 106 laboratories in Italy. They concluded that there is a high variability in the results (15). 

Our results were better from Brooks who indicated a significant lack of standardization in the performance and reporting of semen analysis among laboratories in the United States. They evaluated the level of standardization in performance of the semen analysis among 536 clinical laboratories in the United States. The participant laboratories routinely reported sperm count (94% of laboratories), motility (95%), morphology (85%), forward progression (69%), and semen volume (96%) as part of the semen analysis. Only 64% of the laboratories reported abstinence routinely, and 60% of laboratories indicated the criteria used for the sperm morphology on the report form. Few laboratories performed quality control for sperm counts (29%), motility (41%), and morphology (41%) (16).

Our values correlate fairly well with Riddel and further support the concept of the need for education and training initiatives to encourage laboratories to become compliant with current WHO guidelines for sperm morphology assessment. They conducted a survey of the methods used to undertake the assessment of sperm morphology during semen analysis in 37 laboratories in the UK and found that only two out of the 37 laboratories (5%) were compliant with all WHO guidelines regarding morphology assessment, including methods of staining and observation, classifying and sampling methods, and the participation in internal and external quality control programs (17).

The need for consistency in the performance of semen analysis was a stimulus for the German Society of Andrology (DGA) to establish an external quality control (EQC) program: Qua-DeGA (18). Participation in a quality control program became compulsory in Germany for all laboratories performing semen analysis in 2011. Twice a year, each laboratory participating in QuaDeGA received two tubes containing 0.5 ml fixed semen for the determination of concentration and normal morphology percentage. They have reported a steady increase in the number of participants, from 27 at the first run to 280 in the nineteenth run. An increase (from 10% to 26%, from 5 to 68) was observed in the laboratories following the WHO-recommended sperm counting procedure during the program. However, the opposite occurred for morphology (i.e., staining method and criteria), where percentage adherence decreased from 34% to 16%. They found that less than 8% of QuaDeGA participating laboratories followed the WHO guidelines, and this rate changed a little over the time. Unfortunately, final conclusion of the first years of QuaDeGA was that adherence to WHO recommendations was low, and most of the medical laboratories using methods expressly opposed the guidelines (18).

## Conclusion

Most of the selected medical laboratories in Tehran were following the update instructions in semen analysis. Although adherence to the WHO recommendations in each section is moderate in the performance and reporting, the process needs to be improved by continuing the education of medical laboratory staff and establishment of the internal and external quality control programs. 
